# Prevalence, determinants, and management of chronic kidney disease in Karachi, Pakistan - a community based cross-sectional study

**DOI:** 10.1186/1471-2369-15-90

**Published:** 2014-06-13

**Authors:** Saleem Jessani, Rasool Bux, Tazeen H Jafar

**Affiliations:** 1 Department of Community Health Sciences, Aga Khan University, Karachi, Pakistan; 2 Department of Medicine, Section of Nephrology, Aga Khan University, Karachi, Pakistan; 3 Division of Nephrology, Department of Medicine, Tufts Medical Center, Boston, MA, USA; 4 Health Services & Systems Research, Duke-NUS Graduate Medical School, Singapore, Singapore

**Keywords:** Albuminuria, Chronic kidney disease, CKD-EPI Pakistan, Glomerular filtration rate, South Asians

## Abstract

**Background:**

Chronic kidney disease (CKD) is increasing being recognized as a global public health problem. However, there is dearth of information on the prevalence, determinants, and management of CKD from low- and middle-income countries. The objectives of the study were to determine the 1) prevalence of CKD; 2) socio-demographic and clinical factors associated with CKD; and 3) the existing management of these patients with regards to blood pressure control, and use of antihypertensive medications.

**Methods:**

We conducted a cross-sectional study on 2873 participants aged ≥40 years in 12 representative communities in Karachi, Pakistan. The primary outcome was clinically significant CKD defined as estimated glomerular filtration rate (eGFR) <60 mL/min/1.73 m^2^ estimated by CKD-EPI (CKD Epidemiology Collaboration) Pakistan equation (0.686 × CKD-EPI^1.059^) or urinary albumin to creatinine ratio ≥3 mg/mmol (i.e. KDOQI CKD stage G3, A2 or worse).

**Results:**

The overall prevalence (95% CI) of CKD was 12.5% (11.4 – 13.8%). The factors independently associated with CKD were older age, hypertension, diabetes, elevated systolic blood pressure, raised fasting plasma glucose, raised triglycerides, and history of stroke (p < 0.05 for each). About 267 (74.4%, 69.5 – 78.8%) adults with CKD had concomitant hypertension. Of these, 130 (48.7%, 42.6 – 54.9%) were on antihypertensive medications, and less than 20% had their BP controlled to conventional target of ≤140/90 mm Hg, and only 16.9% (12.6 – 21.9%) were on blockers of renin-angiotensin system alone or in combination with other drugs.

**Conclusions:**

Clinically significant CKD is common among Pakistani adults. The conventional risk factors for CKD and poor control of blood pressure among patients with CKD highlight the need to integrate CKD prevention and management in the primary care infrastructure in Pakistan, and possibly neighbouring countries.

## Background

Chronic kidney disease (CKD) is increasing being recognized as a major public health problem globally [1]. The adverse outcomes associated with CKD including kidney failure, accelerated cardiovascular disease (CVD), and premature mortality have greater societal and economical impact in low- and middle-income countries [2]. A glomerular filtration rate (GFR) level of less than 60 ml/min/1.73 m^2^ (GFR stages G3a – G5), indicating CKD represents loss of half or more of the adult level of normal kidney function, the level below which the risk of adverse outcomes has been shown to increase. As demonstrated in a large meta-analysis of a large general-population cohort of 105,872 participants, albuminuria is an independent marker of increase CVD mortality [3].

The Kidney Disease: Improving Global Outcomes (KDIGO) Clinical Practice Guidelines 2012 for the Evaluation and Management of Chronic Kidney Disease classify CKD based on eGFR stages (G1 through G5 using eGFR thresholds (G3 split G3a and G3b using eGFR threshold of 45 ml/min/1.73 m^2^), and albuminuria stages (A1 (<3 mg/mmol), A2 (3 to 30 mg/mmol) and A3 (>30 mg/mmol)). The guidelines also recommend using locally validated CKD-EPI equation as preferred methods for estimating GFR where available [4].

The burden of CKD may be further exaggerated in rapidly urbanizing South Asian country like Pakistan where a substantial proportion of 180 million are predisposed to chronic diseases including diabetes and hypertension by virtue of low birth weight possibly associated with reduced renal reserve [5]. Moreover, South Asian countries are undergoing an epidemiological transition with an increase in risk factors of CKD, and consequently posing a burden on health systems [6]. Furthermore, CKD is also known to progress fast in Asians compared to Western counterparts underscoring the need for prevention through early detection and management of risk factors [7]. However, there is dearth of representative data on the prevalence and determinants of CKD from South Asian countries including Pakistan. Furthermore, despite publications of clear guidelines regarding the importance of blood pressure (BP), control and trials demonstrating effectiveness of blockers of renin-angiotensin system in patients with CKD, it is not known how these patients are managed in low- and middle-income countries that have traditionally diverted resources for treating acute infectious diseases [8,9].

The objectives of this study were to determine the 1) prevalence of CKD stage G3, A2 or worse; 2) socio-demographic and clinical factors associated with CKD; and 3) the existing management of patients with CKD with regards to BP control, and use of antihypertensive medications among adults in Karachi, Pakistan.

## Methods

### Study setting

This was a cross-sectional study representative of urban city of Karachi, Pakistan, conducted as part of baseline within a factorial design cluster randomized controlled trial [10]. In brief, the Federal Bureau of Statistics has divided the city of Karachi, the most populous city in Pakistan with 18 million inhabitants, into 5000 clusters each of about 250 households each. A multistage random sampling was employed to identify 12 of 4200 low to middle income (mean household monthly income $70) clusters (Additional file 1: Figure S1). All adults aged ≥ 40 years in these households were invited and the written informed consent was obtained from all study participants. Ethical approval was obtained from the Aga Khan University Ethics Review Committee.

### Screening

Trained research staff visited all households in each of the 12 clusters, and informed consent was obtained for screening from all eligible adults; who then underwent for measurement of BP three times. The study was conducted over one year during 2004 – 2005.

A routine physical examination was performed and the following information collected: (i) smoking status, food frequency and physical activity (IPAQ, international physical activity questionnaire), co-morbidities (history of stroke, cardiovascular disease, known diabetes, known hypertension); (ii) anthropometry (height, weight and waist circumference); (iii) BP was measured thrice with a calibrated automated device (Omron HEM-737 IntelliSense; Omron Healthcare Inc., Vernon Hills, IL) in the sitting position after 5 minutes of rest. If BP was elevated (systolic blood pressure (SBP) ≥140 mm Hg or diastolic blood pressure (DBP) ≥ 90 mm Hg) based on mean of last 2 of 3 readings, subjects were visited again after 1 – 4 weeks for re-measurement of BP to confirm hypertension status if BP were persistently elevated, and (iv) Blood specimens for glucose, creatinine and lipids profile were collected in the morning after an overnight fast of about 10 – 12 hours. For urine microalbumin and creatinine, first morning sample was collected. All samples were transported in about one hour to the clinical laboratory at the Aga Khan University Hospital where samples were processed and stored at 2 – 8°C for appropriate tests performed within 24 hours. Serum creatinine and fasting blood glucose were measured on Synchron Cx-7/Delta; Beckman Coulter, Fullerton, CA; the lipid profile was measured on Hitachi-912; Roche, Basel, Switzerland. Urine microalbumin and creatinine were measured using nephelometry by the Array Systems method on a Beckman Coulter and Synchron Cx-7/Delta. All tests were performed to a standard protocol that confirmed to the international standards for definitions and measurements.

Serum creatinine measurements were calibrated at the Cleveland Clinic laboratory-reference laboratory, where serum creatinine levels were measured again using the Roche enzymatic creatinine assay (in duplicate) which is traceable to the National Institute of Standards and Technology creatinine reference measurement [11].

### Variable definitions

Glomerular Filtration Rate (GFR) was estimated using the CKD-EPI (CKD Epidemiology Collaboration) Pakistan (CKD-EPI_PK_) equation, a modified version of CKD-EPI creatinine equation with a correction factor (0.686 × CKD-EPI^1.059^) for South Asians (estimated GFR (eGFR) based on this equation denoted as eGFR_CKD-EPI(PK)_) [11].

Hypertension was defined as persistent elevation of SBP ≥140 mm Hg or DBP ≥90 mm Hg on the basis of average of last two of three readings measured 5 minutes apart at each visit, on two separate occasions, or taking antihypertensive medications.

Diabetes was defined as fasting blood glucose ≥7.0 mmol/L, or taking anti-diabetic medications.

CKD (stage G3, A2 or worse) was defined as eGFR_CKD-EPI(PK)_ < 60 mL/min/1.73 m^2^ (reduced eGFR) or urinary albumin to creatinine ratio (UACR) ≥3 mg/mmol (albuminuria) based on a single spot urine sample.

### Statistical analysis

The analyses were performed in IBM SPSS v20 and Stata release 12.1 statistical software. The descriptive statistics were performed on 2873 participants by CKD status. Differences in means and proportions were assessed by t-test and chi-square test respectively. The distribution of eGFR in men and women was assessed graphically by plotting median, 25^th^ and 75^th^ percentiles respectively using quantile regression. The crude and age-standardized prevalence (95% CI) of CKD was computed. The reference population for the latter was WHO (World Health Organization) World Standard Population [12].

The final sample size for multivariable models was 2823 because of missing information on hypertension status on 50 subjects (Additional file 2: Figure S2). Two multivariable models were built for primary outcome of CKD by stepwise logistic regression analysis using forward selection procedure with an entry criteria of p = 0.05 and removal criteria p = 0.20. The first model was based on socio-demographic determinants (age, sex, education, tobacco use, and physical activity), and the second model was based on socio-demographic plus clinical predictors, i.e. hypertension, diabetes, self-reported history of coronary heart disease, self-reported history of stroke, body mass index (BMI), fasting plasma glucose, serum cholesterol, high density lipoprotein cholesterol (HDL), low density lipoprotein cholesterol (LDL), and serum triglycerides. Both the multivariable models developed using stepwise logistic regression were adjusted for clustering; and furthermore, second model was also adjusted for co-morbidities, i.e. history of coronary heart disease and history of stroke.

Moreover, all CKD patients with hypertension were also assessed for 1) control of BP to levels ≤ 130/80 mm Hg, and ≤140 /90 mm Hg, and 2) use of anti-hypertensive medications by UACR status, i.e. UACR <3 mg/mmol and ≥3 mg/mmol, respectively. For these analyses, KDIGO 2012 Clinical Practice Guideline based disease-specific definition of hypertension in CKD (BP >140/90 or BP >130/80 mm Hg for those with UACR <3 mg/mmol and ≥3 mg/mmol, respectively) was used [8]. The use of antihypertensive medications by drug classes was assessed. Single drugs used alone represented mono-therapy and counted as individual drug; whereas two or more single drugs taken together either separately or as fixed dose combination drugs were counted under combination category (and not in the individual drug categories). The p-value <0.05 was considered statistically significant.

## Results

A total of 3143 adults were aged 40 years or older in the 12 communities, and were invited for blood and urine tests; these measurements were available on 2873 (91.4%) individuals. The baseline characteristics of the population with and without CKD are shown in Table 1. Moreover, the baseline characteristics according to eGFR and UACR categories are shown in the Additional file 3: Tables S1 and S2.

**Table 1:** Socio-demographic and clinical characteristics of individuals with and without Chronic Kidney Disease

**Characteristics**	**Total (n = 2873)**	**No CKD 2514 (87.4)**	CKD* 359 (12.5)	**P-value**
Age in years, mean ± SD	51.5 ± 10.7	50.5 ± 10.0	58.8 ± 12.3	<0.001
Women, n (%)	1499 (52.2)	1299 (51.7)	200 (55.7)	0.152
Education, n (%)				
No education	992 (34.5)	832 (33.1)	160 (44.6)	<0.001
Primary & middle	929 (32.3)	813 (32.3)	116 (32.3)	
Secondary & higher secondary	622 (21.6)	565 (22.5)	57 (15.9)	
Graduate and above	330 (11.5)	304 (12.1)	26 (7.2)	
Tobacco use, n (%)				
Current users	1113 (38.7)	980 (39.0)	133 (37.0)	0.003
Past users	280 (9.7)	227 (9.0)	53 (14.8)	
Never users	1480 (51.5)	1307 (52.0)	173 (48.2)	
Employed in any occupation, n (%)	1209 (42.1)	1115 (44.4)	94 (26.2)	<0.001
Physical Activity, METs < 840, n (%)	1725 (60.0)	1473 (58.6)	252 (70.2)	<0.001
Hypertension, n (%)║^§^	1267 (44.9)	1013 (41.0)	254 (72.2)	<0.001
Diabetes Mellitus, n (%)^†^	615 (21.4)	465 (18.5)	150 (41.8)	<0.001
History of CHD, n (%)^††^	246 (8.6)	203 (8.1)	43 (12.0)	0.013
History of stroke, n (%)	88 (3.1)	59 (2.3)	29 (8.1)	<0.001
Weight in Kg, mean ± SD	64.8 ± 14.4	65.0 ± 14.3	63.4 ± 14.6	0.063
Body mass index, mean ± SD║	25.8 ± 5.5	25.8 ± 5.5	25.7 ± 5.1	0.767
Systolic BP, mean ± SD	137 ± 24	135 ± 22	153 ± 27	<0.001
Diastolic BP, mean ± SD	86 ± 13	85 ± 12	91 ± 15	<0.001
Fasting plasma glucose, mean ± SD^‡^	6.4 ± 2.8	6.2 ± 2.6	7.7 ± 3.8	<0.001
Serum Cholesterol, mean ± SD^‡^	4.9 ± 1.0	4.8 ± 1.0	5.0 ± 1.2	0.003
LDL, mean ± SD^‡^	3.0 ± 0.8	3.0 ± 0.8	3.1 ± 1.0	0.087
HDL, mean ± SD^‡^	1.0 ± 0.3	1.0 ± 0.3	1.1 ± 0.3	0.196
Triglycerides, mean ± SD^‡^	1.8 ± 1.1	1.8 ± 1.1	2.0 ± 1.2	<0.001
eGFR, median (25^th^ –75^th^ percentiles)	92.8 (82.7 – 101.5)	94.6 (85.9 – 102.1)	68.2 (52.1 – 92.5)	0.001
UACR, median (25^th^ –75^th^ percentiles)	0.58 (0.38 – 1.07)	0.53 (0.36 – 0.83)	6.24 (3.46 – 19.60)	<0.001

CKD = Chronic Kidney Disease; METs = Metabolic Equivalents; CHD = Coronary Heart Disease; BP = Blood Pressure; LDL = Low Density Lipoprotein cholesterol; HDL = High Density Lipoprotein cholesterol; UACR = Urinary Albumin to Creatinine Ratio.

*CKD is defined as the eGFR < 60 mL/min/1.73 m^2^ estimated by CKD-EPI_PK_ (0.686 × CKD-EPI^1.059^) or urinary albumin to creatinine ratio (UACR) ≥3 mg/mmol.

║Missing observations: for hypertension status 50 were missing and for BMI 2 were missing.

^§^Hypertension was defined as persistent elevation of SBP ≥140 mm Hg or DBP ≥90 mm Hg on the basis of average of last two of three readings measured 5 minutes apart at each visit, on two separate occasions, or taking antihypertensive medications.

^†^Diabetes defined as fasting blood glucose ≥ 7.0 mmol/L or on anti-diabetic medications.

^††^CHD was defined as self-reported history of coronary heart disease.

^‡^Reported in SI units (mmol/L).

### Missing information

Information on serum creatinine and UACR measurements was missing in 261 (8.3%), and 253 (8.0%) participants respectively. The characteristics of subjects with (n = 270) and without missing information on CKD (n = 2873) (i.e. serum creatinine and/or UACR) did not differ by age (51.5 vs. 52.6 years, p = 0.14), gender (men, 48 vs. 48%, p = 0.99), SBP (137 vs. 137 mm Hg, p = 0.576) or history of stroke (3.1 vs. 3.7%, p = 0.563). However, mean BMI (25.8 vs. 24.1 Kg/m^2^, p < 0.001), mean DBP (86 vs. 84 mm Hg, p = 0.024) or proportion of hypertension (44.9 vs. 32.9%, p < 0.001) was higher among subjects with available CKD information compared to those with missing information. An additional 50 individuals did not have hypertension status confirmed. A comparison of the above-mentioned characteristics with the individuals with complete information (n = 2,823) did not show any significant difference with respect to age, gender, SBP, DBP, or stroke.

### CKD prevalence

**Figure 1:**
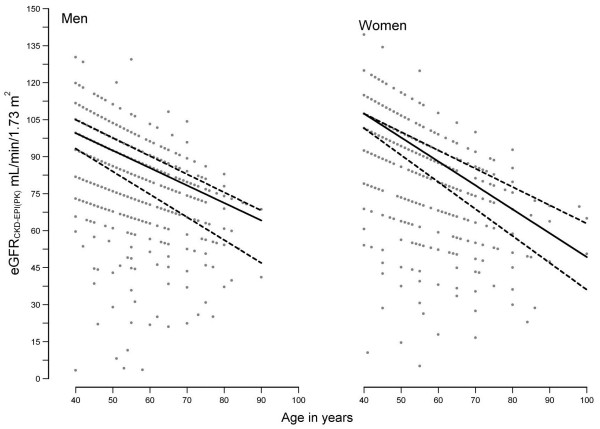
**eGFR**_**CKD-EPI(PK) **_**distribution by age in men and women.** The lines have been plotted using quantile regression in Stata. The solid line represent median eGFR and dashed lines represents 25^th^ and 75^th^ percentiles respectively. Abbreviation: eGFR_CKD-EPI(PK),_ eGFR calculated by CKD-EPI Pakistan (0.686 × CKD-EPI^1.059^).

The distribution of eGFR among adult men and women is illustrated in Figure 1. The crude prevalence (95% CI) of reduced eGFR (eGFR_CKD-EPI(PK)_ <60.0 mL/min/1.73 m^2^), albuminuria (UACR ≥3 mg/mmol), and CKD was 5.3% (4.5 – 6.2%), 9.4% (8.4 – 10.5%), and 12.5% (11.4 – 13.8%) respectively.

**Figure 2:**
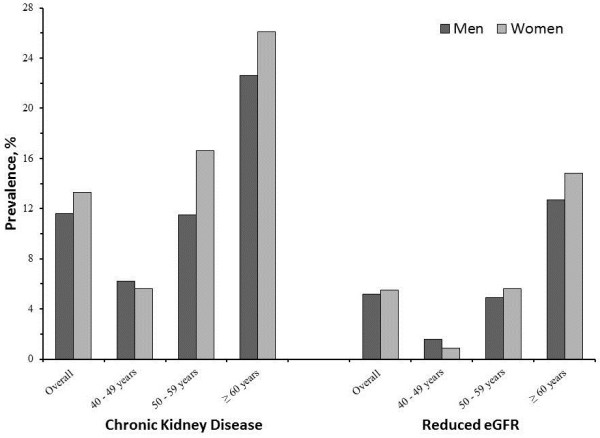
**Age and sex specific crude prevalence of chronic kidney disease and reduced eGFR.** The dark and light grey bars represent men and women respectively. The prevalence percentages are plotted on y-axis and the age groups are plotted on the x-axis.

The prevalence of CKD and reduced eGFR was higher in women compared to men, and increased with age (Figure 2). The age standardized prevalence (95% CI) of reduced eGFR (eGFR_CKD-EPI(PK)_ <60.0 mL/min/1.73 m^2^), albuminuria (UACR ≥3 mg/mmol), and CKD was 7.4% (6.2 – 8.6%), 11.1% (9.8 –12.4%), and 15.3% (13.7 – 16.9%) respectively.

### CKD determinants

Among the 2873 adults, a total of 359 (12.5%) had CKD. These patients were significantly older, more likely to be less physically active, have concomitant hypertension, diabetes, coronary heart disease and stroke compared to those without CKD (p < 0.05 for each).

Table 2 shows adjusted odds ratios and 95% CI of factors associated with CKD. The socio-demographic and clinical factors independently associated with presence of CKD were older age, hypertension, diabetes, elevated SBP, raised fasting plasma glucose, raised triglycerides, and history of stroke, (p < 0.05 for each)

**Table 2:** Multivariable regression models for Chronic Kidney Disease

**Characteristics**	Model 1*^† ^**Adjusted OR (95% CI)**	Model 2*^‡ ^**Adjusted OR (95% CI)**
Age in years	1.35 (1.28 – 1.41) For each 05 year increase	1.31 (1.24 – 1.38) For each 05 year increase
Physical activity		
< 840 METs	1.35 (1.04 – 1.75)	-
≥ 840 METs	1.00	
Hypertension		
Hypertensive	NA	1.90 (1.40 – 2.57)
Non-hypertensive		1.00
Diabetes mellitus		
Diabetic	NA	1.69 (1.18 – 2.43)
Non-diabetic		1.00
Systolic BP, mm Hg	NA	1.15 (1.09 – 1.22) For each 10 mm Hg increase
Fasting plasma glucose, mmol/L	NA	1.08 (1. – 1.13) For each 1 mmol/L increase
Triglycerides, mmol/L	NA	1.07 (1.01 – 1.13) For each 0.5 mmol/L increase
History of stroke		
Positive	NA	1.73 (1.03 – 2.92)
Negative		1.00

NA = Not applicable; METs = Metabolic Equivalents; BP = Blood Pressure.

*Final sample size for multivariable models was 2823 and models are based on stepwise forward selection method.

^†^The candidate variables for model 1 include socio-demographic determinants only, i.e. age, gender, educational status, tobacco use and physical activity. The final model was adjusted for clustering.

^‡^The candidate variables for model 2 include all socio-demographic and clinical variables, i.e. age, gender, educational status, tobacco use and physical activity, hypertension status, diabetes status, history of CHD, history of stroke, body mass index, systolic BP, diastolic BP, fasting plasma glucose, serum cholesterol, low density lipoprotein cholesterol, high density lipoprotein cholesterol, and total triglycerides. The final model was adjusted for clustering and history of coronary heart disease.

### CKD and hypertension

Among 359 individuals with CKD, 267 (74.4%, 95% CI: 69.5 – 78.8%) had concomitant hypertension using CKD specific definition. About 48.7% (42.6 – 54.9%) of these patients were on antihypertensive medications, and less than 20% had their BP controlled to conventional target of ≤140/90 mm Hg (Table 3). Beta blockers were the single most commonly prescribed antihypertensive agent (16.1%, 95% CI: 11.9 – 21.1), followed by various combinations of antihypertensive medications (13.5%, 95% CI: 9.6 – 18.2). Only 9.7% (6.5 – 13.9%) were on blockers of renin-angiotensin system alone whereas 16.9% (12.6 – 21.9%) patients were using these medications either as mono-therapy or in combination with other antihypertensive drugs (Table 4).

**Table 3:** Blood pressure control and use of antihypertensive medication among CKD patients with hypertension according to level of UACR

**Characteristics**	**Overall, n (%, 95% CIs)**	**UACR, mg/mmol, n (%, 95% CIs)**		
		**<3.0**	**≥ 3.0**	**≥3.0 and diabetes**
	**n = 267**	**N = 56**	**N = 211**	**N = 107**^**†**^
BP ≤ 140/90 mm Hg, n (%, 95% CI)	51 (19.1, 14.6 – 24.3)	13 (23.2, 13.0 – 36.4)	38 (18, 13.1 – 23.9)	20 (18.7, 11.8 – 27.4)
BP ≤ 130/80 mm Hg, n (%, 95% CI)	17 (6.4, 3.8 – 10.0)	6 (10.7, 4.0 – 21.9)	11 (5.2, 2.6 – 9.1)	6 (5.6, 2.1 – 11.8)
Use of antihypertensive medications, n (%, 95% CI)	130 (48.7, 42.6 – 54.9)	33 (58.9, 45.0 – 71.9)	97 (46, 39.1 – 52.9)	54 (50.5, 40.6 – 60.3)

UACR = Urinary Albumin to Creatinine Ratio; BP = Blood Pressure.

CKD (G3, A2 or worse) is defined as the eGFR < 60 mL/min/1.73 m^2^ estimated by CKD-EPI_PK_ equation (0.686 × CKD-EPI^1.059^) or UACR ≥3 mg/mmol.

^†^Patients with diabetes mellitus and UACR ≥3 mg/mmol along with CKD and hypertension.

**Table 4:** Use of antihypertensive medication by class of drugs among CKD patients with hypertension according to level of UACR

**Anti-hypertensive medications**	**Overall, n (%, 95% CIs)**	**UACR, mg/mmol, n (%, 95% CIs)**		
		**<3.0**	**≥3.0**	**≥3.0 and diabetes**
	**n = 267**	**N = 56**	**N = 211**	**N = 107**^**‡**^
Antihypertensive medications, mean ± SD	1.4 ± 0.8	1.4 ± 0.7	1.4 ± 0.8	1.5 ± 0.9
ACEI/ARB only	26 (9.7, 6.5 – 13.9)	7 (12.5, 5.2 – 24.1)	19 (9.0, 5.5 – 13.7)	10 (9.3, 4.6 – 16.5)
ACEI/ARB only or in combination with others	45 (16.9, 12.6 – 21.9)	12 (21.4, 11.6 – 34.4)	33 (15.6, 11–21.3)	20 (18.7, 11.8 – 27.4)
Beta blockers only	43 (16.1, 11.9 – 21.1)	9 (16.1, 7.6 – 28.3)	34 (16.1, 11.4 – 21.8)	13 (12.1, 6.6 – 19.9)
Beta blockers only or in combination with others	64 (24.0, 19.0 – 29.6)	15 (26.8, 15.8 – 40.3)	49 (23.2, 17.7 – 29.5)	22 (20.6, 13.4 – 29.5)
Calcium channel blockers only	18 (6.7, 4.0 – 10.4)	4 (7.1, 2.0 – 17.3)	14 (6.6, 3.7 – 10.9)	10 (9.3, 4.6 – 16.5)
Calcium channel blockers only or in combination with others	40 (15.0, 10.9 – 19.8)	9 (16.1, 7.6 – 28.3)	31 (14.7, 10.2 – 20.2)	21 (19.6, 12.6 – 28.4)
Diuretics only	3 (1.1, 0.2 – 3.2)	2 (3.6, 0.4 – 12.3)	1 (0.5, 0.0 – 2.6)	1 (0.9, 0.0 – 5.1)
Diuretics only or in combination with others	19 (7.1, 4.3 – 10.9)	6 (10.7, 4–21.9)	13 (6.2, 3.3 – 10.3)	12 (11.2, 5.9 – 18.8)
Any combinations^†^	36 (13.5, 9.6 – 18.2)	10 (17.9, 8.9 – 30.4)	26 (12.3, 8.2 – 17.5)	18 (16.8, 10.3 – 25.3)

UACR = Urinary Albumin to Creatinine Ratio; ACEI/ARB = Angiotensin converting enzyme inhibitor/Angiotensin receptor blocker.

^†^Combination antihypertensive medications are based on various combinations of 2 or more above-mentioned anti-hypertensive drugs. They are not included in the individual drug categories. Each individual drug refers to monotherapy.

^‡^Patients with diabetes mellitus and UACR ≥3 mg/mmol along with CKD and hypertension.

## Discussion

Our population based study is the first to report prevalence and factors associated with CKD from a South Asian country using a locally validated eGFR equation. Our findings indicate a high (12.5%) prevalence of CKD, defined using validated eGFR_CKD-EPI(PK)_ <60 mL/min/1.73 m^2^, and/or albuminuria ≥3 mg/mmol or higher, among adults aged 40 years or older in Karachi, Pakistan. The prevalence of reduced eGFR_CKD-EPI(PK)_ alone was 5.3% (4.5 – 6.2%). We found that CKD was independently associated with older age, hypertension, diabetes, elevated SBP, raised fasting plasma glucose, raised triglyceride levels, and history of stroke (p < 0.05 for each). Despite a high proportion of associated co-morbidities, CKD remains under-treated with less than 10% and 20% of patients had their BP controlled to targets of ≤130/80 mm Hg, and ≤140/90 mm Hg, respectively per KDIGO 2012 Clinical Practice guidelines [8]. Our results of high prevalence of CKD underscore urgent need for efforts to prioritize CKD on the public health agenda of Pakistan.

The distribution of eGFR in this population illustrate an age related decline, (Figure 1) albeit with relatively well persevered median eGFR among men and women aged 60 years or older [13]. These findings underscore the significance of low eGFR values which should prompt further evaluation even in the elderly in this population.

The high prevalence of CKD is not surprising given the high burden of major CKD risk factors in South Asia. Hypertension and diabetes, both established risk factors for end stage kidney disease, were independently associated with CKD in this population [14,15]. Results of national surveys indicate that hypertension and diabetes affect about 1 in 3 and 1 in 5 adults, respectively, in Pakistan [16-18]. While, trends data on CKD are not available, the 2010 Global Burden of Disease for Pakistan reported a steep rise in the prevalence of major CKD risk factors during the past two decades [6,19,20]. Our finding of high prevalence of CKD is highly suggestive of a parallel increase in the neglected burden of CKD in Pakistan.

We observed that high triglyceride levels were independently associated with CKD. These findings are consistent with those in the Western population demonstrating raised triglycerides in individuals with CKD, [21] which in part confers increased cardiovascular risk. We also found patients with CKD were more likely to have concomitant stroke (adjusted OR, 95% CI: 1.73 (1.03 – 2.92). A number of studies have established that both reduced eGFR and urine albumin excretion, even in the high normal range, predict a graded increase in cardiovascular morbidity [3]. Furthermore, the adverse environmental exposures including high levels of ambient air pollutants and heavy metals are likely to further enhance the CVD risk associated with CKD in this population [22]. Although lead was phased out of gasoline in Pakistan in 2002, the chronic exposure would predispose this population to lead-related nephrotoxicity especially in the presence of CKD, hypertension or diabetes [23-25].

Recent trials data suggest benefit of lipid lowering on CVD morbidity and mortality among patients with CKD [26]. This practice needs to be integrated into CKD prevention efforts in Pakistan.

We found that BP control was grossly sub-optimal with less than 20% of patients having BP controlled to conventional target of ≤140/90 mm Hg. The recent 2012 KDIGO CKD Clinical Practice guidelines underscore BP target of 130/80 mm Hg or less if the patient has CKD with a higher degree of albuminuria (UACR ≥3 mg/mmol) [8]. Clearly the vast majority of patients with CKD failed to meet even the more relaxed target of ≤140/90 mm Hg in Pakistan (Table 3). These findings call for enhancing provider and patient education regarding importance of BP control among patients with CKD.

Evidence suggests benefit of antihypertensive therapy and blockers of renin-angiotensin system are especially protective in patients with CKD and albuminuria [27]. Strikingly, despite significant co-morbidities among patients with CKD, only 48.3% (95% CI: 42.6 – 54.9%) received antihypertensive medications. Moreover use of blockers of renin-angiotensin system was grossly inadequate with barely 17% of patients taking these nephro-protective agents [28] (Table 4). Our results highlight the sub-optimal delivery of CKD care in urban Pakistan where private physicians are the dominant source of service provision, and cost of medications is borne out of pocket [6]. Our previous survey of physicians in Pakistan identified serious deficiencies in knowledge and management of hypertension [29]. The situation is likely to be worse for CKD. Studies in the West have shown that screening for CKD can improve BP control among patients recognized to have CKD [30]. Thus, our findings underscore implementation of KDIGO guidelines for screening and management of CKD to be integrated along with management of hypertension and diabetes in the primary health infrastructure in Pakistan, coupled with provider training and public education on awareness of CKD. Appropriate referral mechanism need to be established for those with advanced stages of CKD. Such a model is likely to be cost-effective for prevention of CKD in Pakistan.

Our study has limitations. First, the cross-sectional design of the study does not permit conclusions regarding causation, and reverse causal association of CKD with BP, lipids, and other factors remains a possibility. However, evidence on the importance of controlling raised BP and glucose levels for slowing progression of CKD is well established. Second, since the study was conducted in Karachi, some variation in findings would be expected in other urban and rural areas of Pakistan. However, national survey indicates a high prevalence of hypertension and diabetes across urban and rural Pakistan [16]. Moreover, practices of providers in terms of management of CKD risk factors, and provision of health services in other areas of Pakistan are not better than in Karachi [29]. Thus, prevalence of CKD is also likely to be comparable and our findings generalizable. Third, we relied on single measurement of serum creatinine and UACR, whereas the clinical definition of CKD requires persistent decrease in eGFR or elevation in UACR for at least 3 months. However, single measurement is considered appropriate for epidemiological research, and has been used widely in other studies [31].

The study has several strengths. This is the first report of prevalence of CKD using a validated eGFR equation with a correction developed in the local South Asian population. Moreover, IDMS (Isotope Dilution Mass Spectrophotometry)-traceable serum creatinine, door to door survey and representative population based on census data of Karachi, and high response rate are the main strength of this study. Thus, our findings would be generalizable to the general population.

## Conclusions

In summary, our findings are the first report of prevalence of CKD in Pakistan using a validated CKD-EPI_PK_ estimating equation (eGFR_CKD-EPI(PK)_ <60 mL/min/1.73 m^2^), or albuminuria (UACR ≥3 mg/mmol). We found that a high prevalence of CKD affecting 12.5% of adults aged 40 years or older. CKD was independently associated with older age, hypertension, diabetes, raised systolic BP, raised plasma fasting glucose, raised triglycerides, and history of stroke. Less than 20% of patients with CKD had BP controlled to conventional levels of ≤140/90 mm Hg. Our findings underscore the urgent need for integrating CKD prevention efforts in the primary care infrastructure in Pakistan, and possibly neighbouring countries with high burden of CKD.

## Abbreviations

CKD: Chronic kidney disease; CVD: Cardiovascular disease; BP: Blood pressure; IPAQ: International physical activity questionnaire; SBP: Systolic blood pressure; DBP: Diastolic blood pressure; GFR: Glomerular Filtration Rate; CKD-EPI: CKD Epidemiology Collaboration; CKD-EPI_PK_: CKD-EPI Pakistan; eGFR: Estimated GFR; eGFR_CKD-EPI(PK)_: eGFR estimated by CKD-EPI Pakistan; UACR: Urinary albumin to creatinine ratio; BMI: Body mass index; HDL: High density lipoprotein cholesterol; LDL: Low density lipoprotein cholesterol; KDIGO: (Kidney Disease Improving Global Outcomes); IDMS: Isotope Dilution Mass Spectrophotometry.

## Competing interests

The authors declare that they have no competing interests.

## Authors’ contribution

THJ conceptualized the analytic plan for the study. SJ analyzed the data along with RB and prepared the first draft with advice from THJ. All authors critically reviewed the manuscript and provided input to the interpretation of findings. All authors have read and approved the final manuscript. THJ is the guarantor.

## Pre-publication history

The pre-publication history for this paper can be accessed here:

http://www.biomedcentral.com/1471-2369/15/90/prepub

## Supplementary Material

Additional file 1: Figure S1 Map of Karachi city with randomized study areas. This map showing the randomized study areas marked as (X) has been adapted from town maps published by the “Master Plan Group of Offices, City District Government, Karachi, Year 2002” publically available on URL: http://www.kmc.gos.pk/Contents.aspx?id=94. Click here for file

Additional file 2: Figure S2 Flow diagram of study participants aged ≥ 40 years. Click here for file

Additional file 3: Table S1 Socio-demographic and clinical characteristics of individuals according to levels of estimated glomerular filtration rate. **Table S2.** Socio-demographic and clinical characteristics of individuals with and without albuminuria. Click here for file

## References

[B1] Levey AS Coresh J Chronic kidney disease Lancet 2012 379 165 180 10.1016/S0140-6736(11)60178-5 21840587

[B2] Gansevoort RT Correa-Rotter R Hemmelgarn BR Jafar TH Heerspink HJ Mann JF Matsushita K Wen CP Chronic kidney disease and cardiovascular risk: epidemiology, mechanisms, and prevention Lancet 2013 382 339 352 10.1016/S0140-6736(13)60595-4 23727170

[B3] Matsushita K van der Velde M Astor BC Woodward M Levey AS de Jong PE Coresh J Gansevoort RT Association of estimated glomerular filtration rate and albuminuria with all-cause and cardiovascular mortality in general population cohorts: a collaborative meta-analysis Lancet 2010 375 2073 2081 20483451 10.1016/S0140-6736(10)60674-5 PMC3993088

[B4] Kidney Disease: Improving Global Outcomes (KDIGO) CKD Work Group KDIGO 2012 Clinical practice guideline for the evaluation and management of chronic kidney disease Kidney inter, Suppl 2013 3 1 150

[B5] Luyckx VA Brenner BM Low birth weight, nephron number, and kidney disease Kidney Int Suppl 2005 68 S68 S77 10.1111/j.1523-1755.2005.09712.x 16014104

[B6] Jafar TH Haaland BA Rahman A Razzak JA Bilger M Naghavi M Mokdad AH Hyder AA Non-communicable diseases and injuries in Pakistan: strategic priorities Lancet 2013 381 2281 2290 10.1016/S0140-6736(13)60646-7 23684257

[B7] Fischbacher CM Bhopal R Rutter MK Unwin NC Marshall SM White M Alberti KG Microalbuminuria is more frequent in South Asian than in European origin populations: a comparative study in Newcastle, UK Diabet Med 2003 20 31 36 10.1046/j.1464-5491.2003.00822.x 12519317

[B8] Kidney Disease: Improving Global Outcomes (KDIGO) Blood Pressure Work Group KDIGO Clinical practice guideline for the management of blood pressure in chronic kidney disease Kidney Inter Suppl 2012 2 337 414

[B9] Baltatzi M Savopoulos C Hatzitolios A Role of angiotensin converting enzyme inhibitors and angiotensin receptor blockers in hypertension of chronic kidney disease and renoprotection. study results Hippokratia 2011 15 27 32 21897755 PMC3139675

[B10] Jafar TH Hatcher J Poulter N Islam M Hashmi S Qadri Z Bux R Khan A Jafary FH Hameed A Badruddin SH Chaturvedi N Community-based interventions to promote blood pressure control in a developing country: a cluster randomized trial Ann Intern Med 2009 151 593 601 10.7326/0003-4819-151-9-200911030-00004 19884620

[B11] Jessani S Levey AS Bux R Inker LA Islam M Chaturvedi N Mariat C Schmid CH Jafar TH Estimation of GFR in South Asians: A study from the general population in Pakistan Am J Kidney Dis 2014 63 49 58 10.1053/j.ajkd.2013.07.023 24074822 PMC4428680

[B12] Ahmad OB Boschi-Pinto C Lopez AD Murray CJ Lozano R Inoue M Age Standardization of Rates: A New WHO Standard. GPE Discussion Paper series no. 31 2001 Geneva: WHO

[B13] Wang X Vrtiska TJ Avula RT Walters LR Chakkera HA Kremers WK Lerman LO Rule AD Age, kidney function, and risk factors associate differently with cortical and medullary volumes of the kidney Kidney Int 2014 85 677 685 10.1038/ki.2013.359 24067437 PMC3943620

[B14] Klag MJ Whelton PK Randall BL Neaton JD Brancati FL Ford CE Shulman NB Stamler J Blood pressure and end-stage renal disease in men N Engl J Med 1996 334 13 18 10.1056/NEJM199601043340103 7494564

[B15] Brancati FL Whelton PK Randall BL Neaton JD Stamler J Klag MJ Risk of end-stage renal disease in diabetes mellitus: a prospective cohort study of men screened for MRFIT. Multiple Risk Factor Intervention Trial JAMA 1997 278 2069 2074 10.1001/jama.1997.03550230045035 9403420

[B16] Jafar TH Levey AS Jafary FH White F Gul A Rahbar MH Khan AQ Hattersley A Schmid CH Chaturvedi N Ethnic subgroup differences in hypertension in Pakistan J Hypertens 2003 21 905 912 10.1097/00004872-200305000-00014 12714864

[B17] Shera AS Rafique G Khwaja IA Ara J Baqai S King H Pakistan national diabetes survey: prevalence of glucose intolerance and associated factors in Shikarpur, Sindh Province Diabet Med 1995 12 1116 1121 10.1111/j.1464-5491.1995.tb00430.x 8750223

[B18] Shera AS Jawad F Maqsood A Prevalence of diabetes in Pakistan Diabetes Res Clin Pract 2007 76 219 222 10.1016/j.diabres.2006.08.011 17005289

[B19] Danaei G Finucane MM Lin JK Singh GM Paciorek CJ Cowan MJ Farzadfar F Stevens GA Lim SS Riley LM Ezzati M National, regional, and global trends in systolic blood pressure since 1980: systematic analysis of health examination surveys and epidemiological studies with 786 country-years and 5.4 million participants Lancet 2011 377 568 577 10.1016/S0140-6736(10)62036-3 21295844

[B20] Danaei G Finucane MM Lu Y Singh GM Cowan MJ Paciorek CJ Lin JK Farzadfar F Khang YH Stevens GA Rao M Ali MK Riley LM Robinson CA Ezzati M National, regional, and global trends in fasting plasma glucose and diabetes prevalence since 1980: systematic analysis of health examination surveys and epidemiological studies with 370 country-years and 2.7 million participants Lancet 2011 378 31 40 10.1016/S0140-6736(11)60679-X 21705069

[B21] Arora P Vasa P Brenner D Iglar K McFarlane P Morrison H Badawi A Prevalence estimates of chronic kidney disease in Canada: results of a nationally representative survey CMAJ 2013 185 E417 E423 10.1503/cmaj.120833 23649413 PMC3680588

[B22] Mansha M Ghauri B Rahman S Amman A Characterization and source apportionment of ambient air particulate matter (PM2.5) in Karachi Sci Total Environ 2012 425 176 183 22154210 10.1016/j.scitotenv.2011.10.056

[B23] Kadir MM Janjua NZ Kristensen S Fatmi Z Sathiakumar N Status of children's blood lead levels in Pakistan: implications for research and policy Public Health 2008 122 708 715 10.1016/j.puhe.2007.08.012 18359052 PMC2494596

[B24] Navas-Acien A Guallar E Silbergeld EK Rothenberg SJ Lead exposure and cardiovascular disease–a systematic review Environ Health Perspect 2007 115 472 482 17431501 10.1289/ehp.9785 PMC1849948

[B25] Ekong EB Jaar BG Weaver VM Lead-related nephrotoxicity: a review of the epidemiologic evidence Kidney Int 2006 70 2074 2084 17063179 10.1038/sj.ki.5001809

[B26] Baigent C Landray MJ Reith C Emberson J Wheeler DC Tomson C Wanner C Krane V Cass A Craig J Neal B Jiang L Hooi LS Levin A Agodoa L Gaziano M Kasiske B Walker R Massy ZA Feldt-Rasmussen B Krairittichai U Ophascharoensuk V Fellstrom B Holdaas H Tesar V Wiecek A Grobbee D de Zeeuw D Gronhagen-Riska C Dasgupta T The effects of lowering LDL cholesterol with simvastatin plus ezetimibe in patients with chronic kidney disease (Study of Heart and Renal Protection): a randomised placebo-controlled trial Lancet 2011 377 2181 2192 10.1016/S0140-6736(11)60739-3 21663949 PMC3145073

[B27] Jafar TH Stark PC Schmid CH Landa M Maschio G de Jong PE de Zeeuw D Shahinfar S Toto R Levey AS Progression of chronic kidney disease: the role of blood pressure control, proteinuria, and angiotensin-converting enzyme inhibition: a patient-level meta-analysis Ann Intern Med 2003 139 244 252 10.7326/0003-4819-139-4-200308190-00006 12965979

[B28] Jafar TH Schmid CH Landa M Giatras I Toto R Remuzzi G Maschio G Brenner BM Kamper A Zucchelli P Becker G Himmelmann A Bannister K Landais P Shahinfar S de Jong PE de Zeeuw D Lau J Levey AS Angiotensin-converting enzyme inhibitors and progression of nondiabetic renal disease. A meta-analysis of patient-level data Ann Intern Med 2001 135 73 87 10.7326/0003-4819-135-2-200107170-00007 11453706

[B29] Jafar TH Jessani S Jafary FH Ishaq M Orakzai R Orakzai S Levey AS Chaturvedi N General practitioners' approach to hypertension in urban Pakistan: disturbing trends in practice Circulation 2005 111 1278 1283 10.1161/01.CIR.0000157698.78949.D7 15769769

[B30] Richards N Harris K Whitfield M O'Donoghue D Lewis R Mansell M Thomas S Townend J Eames M Marcelli D Primary care-based disease management of chronic kidney disease (CKD), based on estimated glomerular filtration rate (eGFR) reporting, improves patient outcomes Nephrol Dial Transplant 2008 23 549 555 18065826 10.1093/ndt/gfm857

[B31] Coresh J Selvin E Stevens LA Manzi J Kusek JW Eggers P Van Lente F Levey AS Prevalence of chronic kidney disease in the United States JAMA 2007 298 2038 2047 10.1001/jama.298.17.2038 17986697

