# Supplementary figures and images for: Prevalence, determinants, and management of chronic kidney disease in Karachi, Pakistan - a community based cross-sectional study
Source: BMC Nephrol. 2014 Jun 13;15:90. doi: 10.1186/1471-2369-15-90 (PMC4065316; doi:10.1186/1471-2369-15-90)

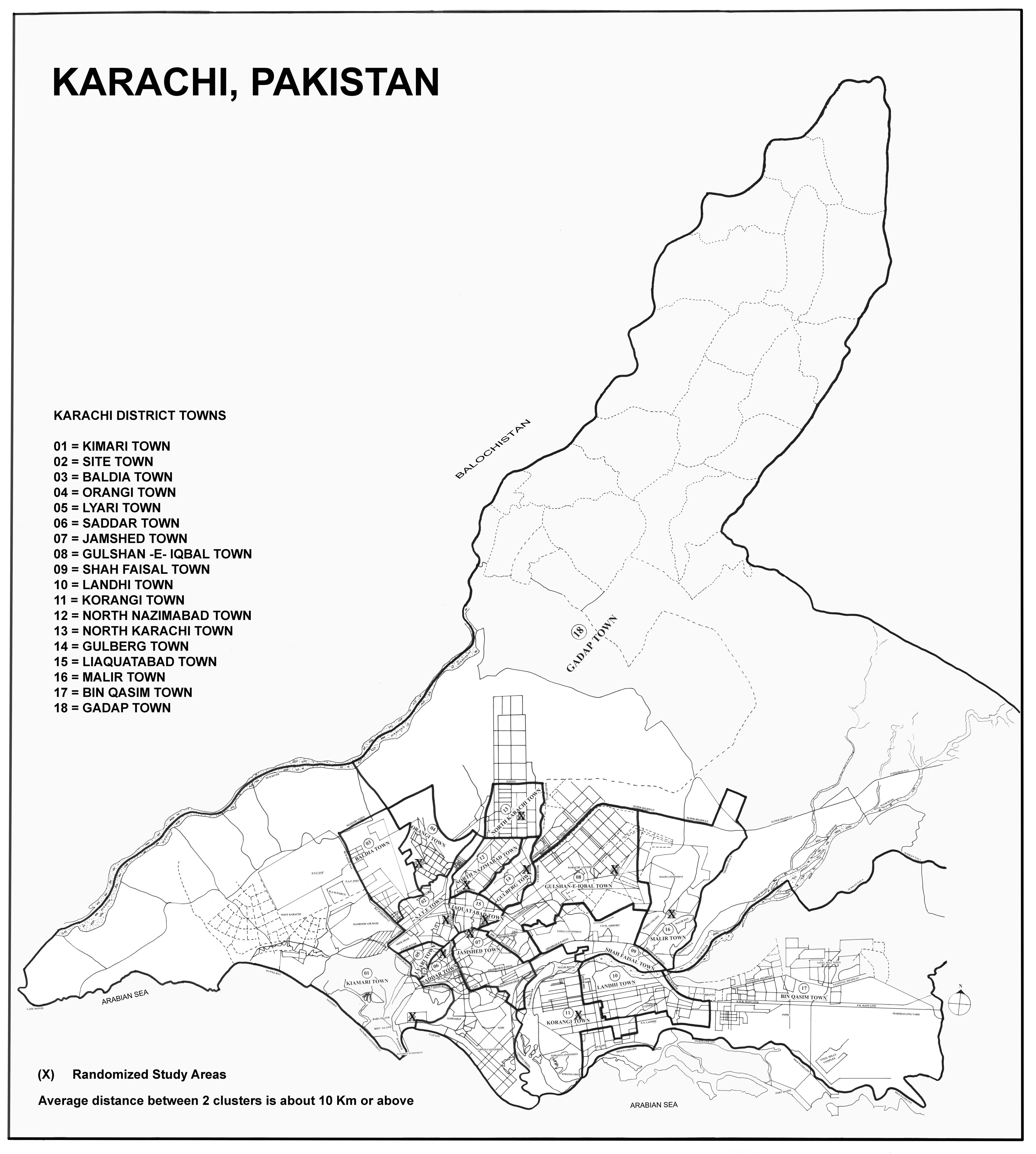

Supplement: Additional file 1: Figure S1 — Map of Karachi city with randomized study areas. This map showing the randomized study areas marked as (X) has been adapted from town maps published by the “Master Plan Group of Offices, City District Government, Karachi, Year 2002” publically available on URL: http://www.kmc.gos.pk/Contents.aspx?id=94. [file 1471-2369-15-90-S1.jpeg]

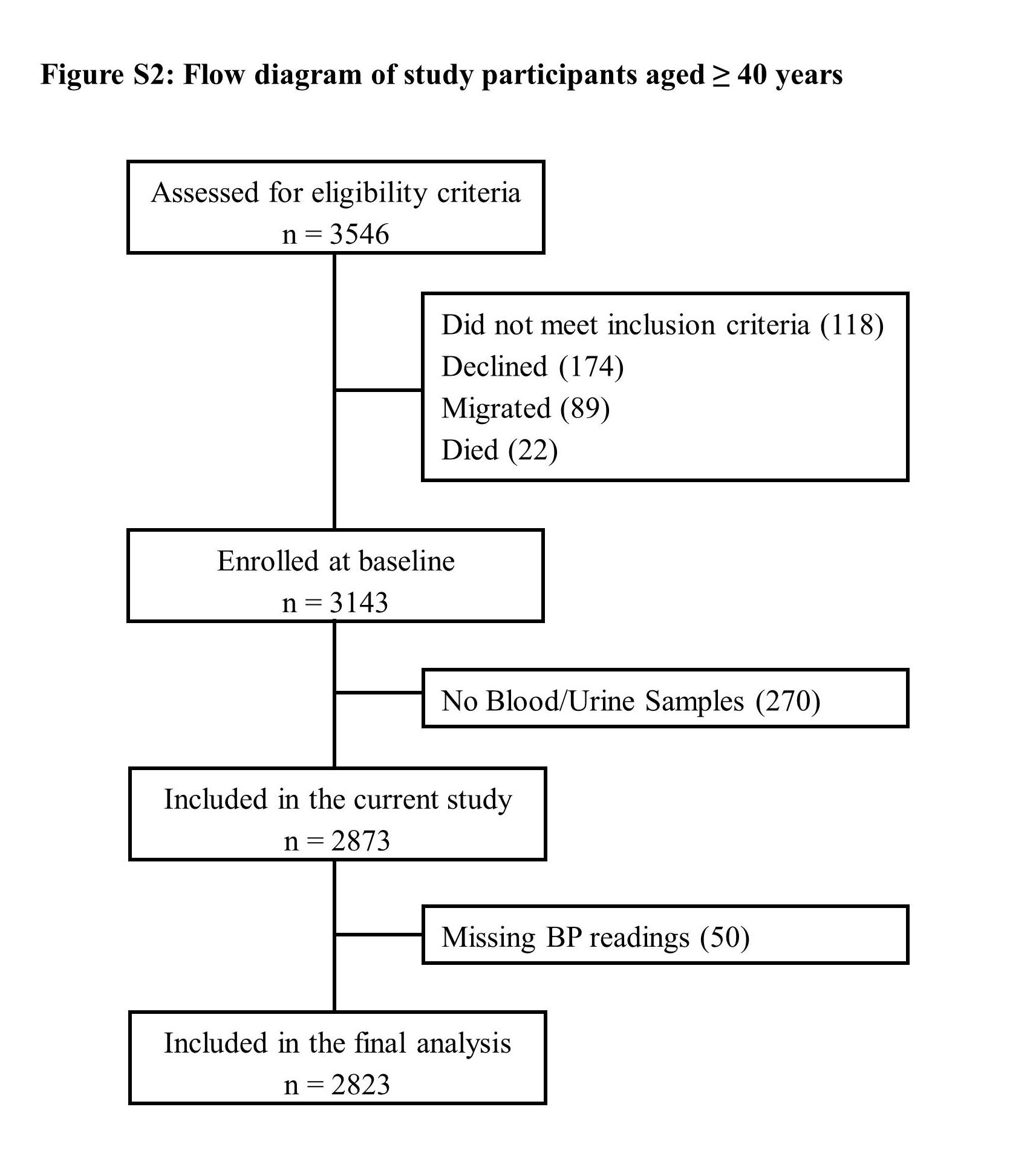

Supplement: Additional file 2: Figure S2 — Flow diagram of study participants aged ≥ 40 years. [file 1471-2369-15-90-S2.jpg]
