# Supplementary material for: Prevalence, determinants, and management of chronic kidney disease in Karachi, Pakistan - a community based cross-sectional study
Source: BMC Nephrol. 2014 Jun 13;15:90. doi: 10.1186/1471-2369-15-90 (PMC4065316; doi:10.1186/1471-2369-15-90)
Supplement: Additional file 3: Table S1 — Socio-demographic and clinical characteristics of individuals according to levels of estimated glomerular filtration rate. Table S2. Socio-demographic and clinical characteristics of individuals with and without albuminuria. [file 1471-2369-15-90-S3.docx]

**Table S1. Socio-demographic and clinical characteristics of individuals according to levels of estimated glomerular filtration rate**

| **Characteristics** | **Estimated Glomerular Filtration Rate mL/min/1.73m^2^** | | | **P-value** |
| --- | --- | --- | --- | --- |
|  | **≥ 90**  **(n =1720)** | **89 – 60**  **(n=1000)** | **< 60**  **(n = 153)** |  |
| Age in years, mean ± SD | 46.4 ± 6.5 | 58.4 ± 11.0 | 63.5 ± 12.4 | <0.001 |
| Women, n (%) | 1009 (58.7) | 408 (40.8) | 82 (53.6) | <0.001 |
| Educational Status, n (%)  No education  Primary & middle  Secondary & higher secondary  Graduate and above | 531 (30.9)  564 (32.8)  412 (24.0)  213 (12.4) | 388 (38.8)  317 (31.7)  187 (18.7)  108 (10.8) | 73 (47.7)  48 (31.4)  23 (15.0)  9 (5.9) | <0.001 |
| Tobacco use, n (%)  Current users  Past users  Never users | 574 (33.4)  120 (7.0)  1026 (59.7) | 486 (48.6)  128 (12.8)  386 (38.6) | 53 (34.6)  32 (20.9)  68 (44.4) | <0.001 |
| Employed in any occupation, n (%) | 754 (43.8) | 426 (42.6) | 29 (19.0) | <0.001 |
| Physical Activity, METs < 840, n (%) | 987 (57.4) | 626 (62.6) | 112 (73.2) | <0.001 |
| Hypertension, n (%)║§ | 673 (39.8) | 482 (49.1) | 112 (74.7) | <0.001 |
| Diabetes Mellitus, n (%)† | 366 (21.3) | 199 (19.9) | 50 (32.7) | 0.002 |
| History of CHD, n (%)†† | 120 (7.0) | 102 (10.2) | 24 (15.7) | <0.001 |
| History of stroke, n (%) | 31 (1.8) | 44 (4.4) | 13 (8.5) | <0.001 |
| Weight in Kg, mean ± SD | 65.9 ± 14.7 | 63.6 ± 13.8 | 60.3 ± 12.3 | <0.001 |
| Body mass index, mean ± SD║ | 26.3 ± 5.7 | 24.9 ± 5.1 | 24.7 ± 4.5 | <0.001 |
| Systolic BP, mean ± SD | 133 ± 21 | 142 ± 25 | 150.27 | <0.001 |
| Diastolic BP, mean ± SD | 86 ± 12 | 86 ± 13 | 87 ± 15 | 0.151 |
| Fasting plasma glucose, mean ± SD‡ | 6.5 ± 2.9 | 6.2 ± 2.6 | 6.7 ± 3.1 | 0.014 |
| Serum Cholesterol, mean ± SD‡ | 4.8 ± 1.0 | 4.9 ± 1.0 | 5.0 ± 1.3 | 0.012 |
| LDL, mean ± SD‡ | 3.0 ± 0.8 | 3.0 ± 0.8 | 3.0 ± 0.8 | 0.342 |
| HDL, mean ± SD‡ | 1.0 ± 0.5 | 1.0 ± 0.5 | 1.0 ± 0.8 | 0.011 |
| Triglycerides, mean ± SD‡ | 1.9 ± 1.1 | 1.8 ± 1.1 | 1.8 ± 0.9 | 0.102 |

CKD = Chronic Kidney Disease; METs = Metabolic Equivalents; CHD = Coronary Heart Disease; BP = Blood Pressure; LDL = Low Density Lipoprotein cholesterol; HDL = High Density Lipoprotein cholesterol

*Estimated Glomerular Filtration Rate was estimated by CKD-EPI_PK_ (CKD-EPI equation with Pakistani correction factor: 0.686 × CKD-EPI^1.059^).

║Missing observations: for hypertension status 50 were missing and for BMI 2 were missing

§ Hypertension was defined as persistent elevation of SBP ≥140 mm Hg or DBP ≥90 mm Hg on the basis of average of last two of three readings measured 5 minutes apart at each visit, on two separate occasions, or taking antihypertensive medications.

†Diabetes defined as fasting blood glucose ≥ 7.0 mmol/L or on anti-diabetic medications.

††CHD was defined as self-reported history of coronary heart disease.

‡ Reported in SI units (mmol/L)

**Table S2. Socio-demographic and clinical characteristics of individuals with and without albuminuria**

| **Characteristics** | **No albuminuria**  **(n = 2603)** | **Albuminuria**  **(n = 270)** | **P-value** |
| --- | --- | --- | --- |
| Age in years, mean ± SD | 51.0 ± 10.4 | 56.7 ± 11.8 | <0.001 |
| Women, n (%) | 1351 (51.9) | 148 (54.8) | 0.362 |
| Educational Status, n (%)  No education  Primary & middle  Secondary & higher secondary  Graduate and above | 878 (33.7)  840 (32.3)  576 (22.1)  309 (11.9) | 114 (42.2)  89 (33.0)  46 (17.0)  21 (7.8) | 0.009 |
| Tobacco use, n (%)  Current users  Past users  Never users | 1013 (38.9)  244 (9.4)  1346 (51.7) | 100 (37.0)  36 (13.3)  134 (49.6) | 0.113 |
| Employed in any occupation, n (%) | 1130 (43.4) | 79 (29.3) | <0.001 |
| Physical Activity, METs < 840, n (%) | 1532 (58.9) | 193 (71.5) | <0.001 |
| Hypertension, n (%)║§ | 1070 (41.8) | 197 (74.3) | <0.001 |
| Diabetes Mellitus, n (%)† | 483 (18.6) | 132 (48.9) | <0.001 |
| History of CHD, n (%)†† | 219 (8.4) | 27 (10.0) | 0.375 |
| History of stroke, n (%) | 63 (2.4) | 25 (9.3) | <0.001 |
| Weight in Kg, mean ± SD | 64.8 ± 14.3 | 64.7 ± 15.3 | 0.935 |
| Body mass index, mean ± SD║ | 25.7 ± 5.5 | 26.0 ± 5.2 | 0.389 |
| Systolic BP, mean ± SD | 135 ± 22 | 155 ± 27 | <0.001 |
| Diastolic BP, mean ± SD | 85 ± 12 | 92 ± 15 | <0.001 |
| Fasting plasma glucose, mean ± SD‡ | 6.2 ± 2.6 | 8.2 ± 4.0 | <0.001 |
| Serum Cholesterol, mean ± SD‡ | 4.8 ± 1.0 | 5.1 ± 1.3 | 0.001 |
| LDL, mean ± SD‡ | 3.0 ± 0.8 | 3.1 ± 1.0 | 0.027 |
| HDL, mean ± SD‡ | 1. 0.3 | 1.0 ± 0.3 | 0.812 |
| Triglycerides, mean ± SD‡ | 1.8 ± 1.3 | 2.1 ± 1.1 | <0.001 |

CKD = Chronic Kidney Disease; METs = Metabolic Equivalents; CHD = Coronary Heart Disease; BP = Blood Pressure; LDL = Low Density Lipoprotein cholesterol; HDL = High Density Lipoprotein cholesterol

*Albuminuria defined as UACR <3.4 mg/mmol on a single spot urine sample.

║Missing observations: for hypertension status 50 were missing and for BMI 2 were missing

§ Hypertension was defined as persistent elevation of SBP ≥140 mm Hg or DBP ≥90 mm Hg on the basis of average of last two of three readings measured 5 minutes apart at each visit, on two separate occasions, or taking antihypertensive medications.

†Diabetes defined as fasting blood glucose ≥ 7.0 mmol/L or on anti-diabetic medications.

††CHD was defined as self-reported history of coronary heart disease.

‡ Reported in SI units (mmol/L)
